# Fat and Dachsous Signaling—Controlling Growth Requires Both Competition and Cooperation: Competitive Interactions at the Cell Cortex Restrict the Ability of an Unusual Protein Complex to Promote Tissue Growth

**DOI:** 10.1002/bies.70103

**Published:** 2026-01-22

**Authors:** Hitoshi Matakatsu, Richard G. Fehon

**Affiliations:** ^1^ Department of Molecular Genetics and Cell Biology The University of Chicago Chicago Illinois USA

**Keywords:** Cell signaling, Protocadherin, Tumor supressor

## Abstract

The Hippo signaling pathway plays a key role in organ size control in normal development and tumorigenesis. While many components of this pathway are well understood, its upstream regulation remains unclear. Among the most enigmatic upstream regulators are the protocadherins Dachsous and Fat. These transmembrane proteins regulate a growth‐promoting complex composed of the atypical myosin Dachs, the adaptor protein Dlish, and the palmitoyltransferase Approximated (together termed the core complex). We propose that by default the core complex promotes growth and that Dachsous and Fat, which previously have been thought to act antagonistically, can also function synergistically to repress core complex function and therefore restrict growth. Understanding the molecular mechanisms underlying Dachsous‐Fat signaling offers insight into how multicellular organisms precisely control organ size.

## Graphical abstract

Dachs, Dlish, and Approximated form a core complex at the junctional cortex that promotes growth (On) by repressing Warts. Activity of this complex is repressed (Off) both by Fat‐mediated degradation and by physical removal from the junctional cortex by Dachsous or Fat. Thus, Dachsous and Fat together repress tissue growth.

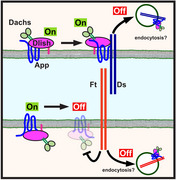

## 1 Introduction

A fundamental question in biology is how the overall size and cell number in an organ are precisely regulated during normal development. For example, in *Drosophila*, a group of about 40 cells in the embryo is specified to form the wing primordium (hereafter referred to as the wing imaginal disc) in the embryo. Wing imaginal disc cells proliferate rapidly during the larval stage, reaching approximately 52 000 cells by the late pupal stage [1, 2]. How is this growth so precisely regulated? Classically, tissue growth during development is thought to be controlled primarily by the morphogen signaling pathways, Decapentaplegic, Hedgehog, Wingless, and Notch, that pattern tissues and drive growth [3]. Additionally, cellular processes, such as cell cycle regulation, metabolic signaling, and apoptosis, can dramatically affect overall tissue growth. Finally, mechanical tension can serve as a cue for tissues to promote uniform growth and perhaps detect overall size [4].

To elucidate the mechanisms controlling tissue growth, genetic screens in *Drosophila* have been used to identify genes that lead to overgrowth when their function is lost, known as tumor suppressors. Among the best known of these are genes in the Hippo signaling pathway [5, 6, 7]. Mutations in the Hippo pathway exhibit a common hyperplastic phenotype, resulting in larger organ size with mostly normal differentiated adult morphology. The core components of the Hippo signaling pathway include kinases (Hippo, Warts (Wts), and Tao‐1) and scaffold proteins such as Mob as a tumor suppressor and Salvador. These kinases are assembled into a complex and activated at the cell cortex by the following two mechanisms: (1) a junctional complex organized by the transmembrane protein Crumbs and the FERM domain protein Expanded (Ex) and (2) a parallel signaling complex assembled in the medial cortex by the WW domain protein Kibra and Merlin, another FERM domain protein [6, 7, 8]. Kinases in the Hippo pathway promote the phosphorylation of the transcriptional co‐activator Yorkie (Yki). Phosphorylated Yki is repressed by preventing its nuclear entry. Once Yki is de‐phosphorylated, it enters the nucleus and promotes the transcription of target genes, including anti‐apoptotic genes, micro‐RNAs, and cell proliferation‐related genes.

**FIGURE 1:**
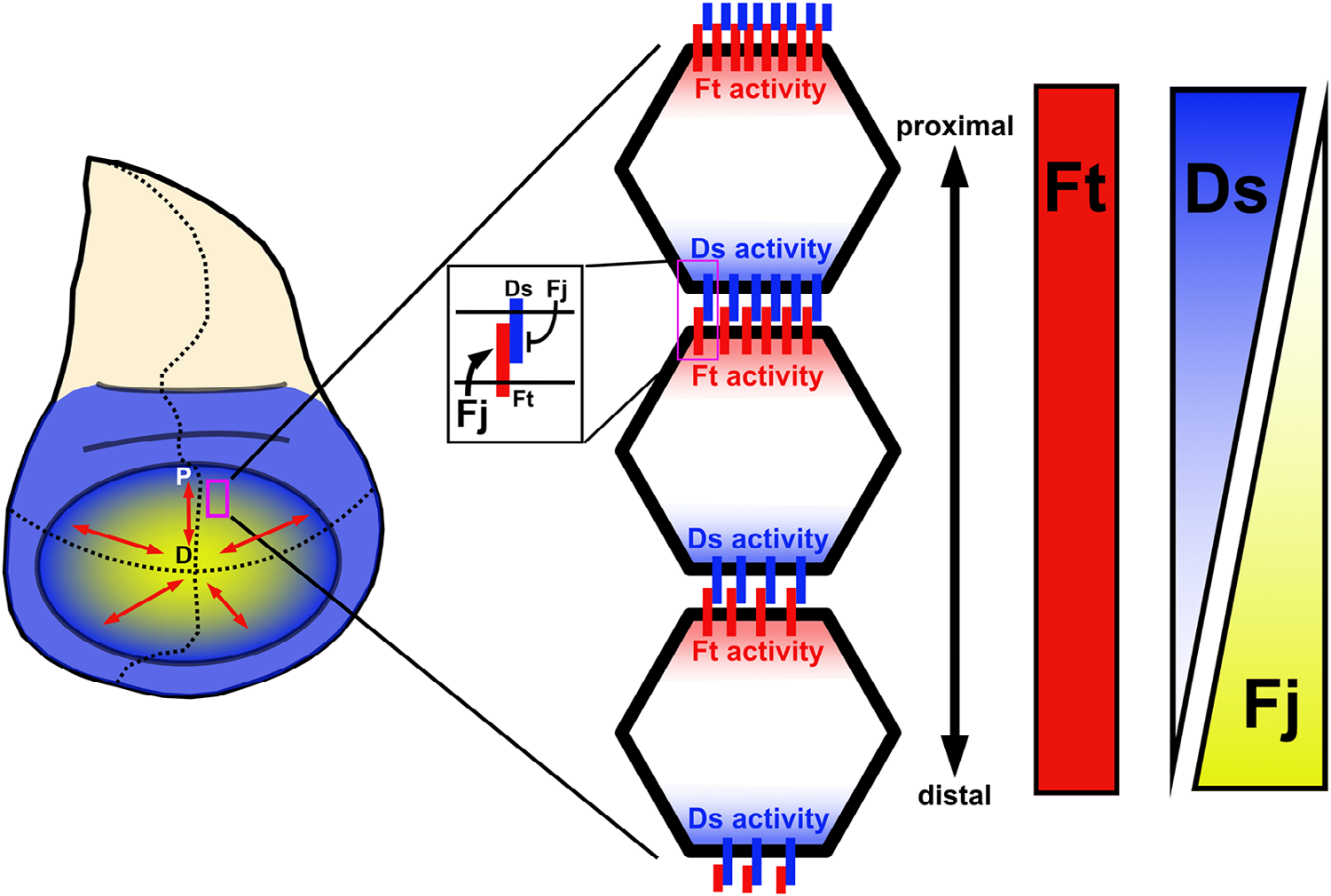
Expression patterns of Ds, Ft, and Fj results in planar polarized activity of Ds and Ft. Ds (blue) is highly expressed in the hinge and proximal wing, but its expression decreases distally. In contrast, Fj (yellow) is highly expressed in the distal wing and reduced in the proximal wing. Ft (red) is expressed uniformly throughout the wing. Fj modulates the binding between Ds and Ft, resulting in a proximal‐distal gradient in binding affinity between Ft and Ds, and planar polarized interactions between these proteins. D, distal; P, proximal.

Among the most enigmatic components of Hippo signaling are Dachsous (Ds) and Fat (Ft), two giant protocadherins that regulate diverse processes including planar cell polarity, patterning, and organ growth [9, 10, 11, 12, 13, 14, 15, 16, 17, 18, 19, 20, 21]. Ft has a large extracellular domain (ECD) with 34 cadherin repeats, EGF‐like domains, laminin‐G domains, and an evolutionarily conserved intracellular domain (ICD) [14, 22]. Similarly, Ds has 27 cadherin repeats and a conserved ICD [15, 22]. Unlike classical cadherins, Ds and Ft preferentially bind heterophilically, acting as a receptor‐ligand pair [23, 24, 25]. Ds and Ft are enriched at the marginal zone and exhibit planar polarization along the body axis in the wing (Figure 1) [24, 25, 26, 27]. The extracellular binding between Ds and Ft is modified by a Golgi‐retained kinase, Four‐jointed (Fj), which phosphorylates the extracellular cadherin domains of Ds and Ft [28, 29, 30]. Phosphorylation by Fj affects Ds and Ft differently: phosphorylation of Ds decreases its binding affinity to Ft, while phosphorylation of Ft by Fj increases its binding affinity to Ds [28, 29]. Opposing proximal‐distal expression gradients of Ds and Fj result in a planar polarized subcellular distribution of Ds on distal junctions and Ft on proximal junctions in the wing imaginal disc (Figure 1). This polarization creates localized activity, with Ds binding and activating Ft at the proximal cell edge [26, 27, 31]. Whether this planar polarized localization of Ft activity is important to growth control is unclear, but it plays a critical role in patterning the tissue.

In addition to its role in planar cell polarity, Ds–Ft signaling is thought to control Hippo pathway output via the atypical myosin Dachs [32, 33]. Previous evidence indicates that Dachs stimulates organ growth by repressing Wts activity [9, 10]. Ft, in turn, suppresses growth by preventing the accumulation of Dachs at the junctional cortex. Additionally, Ft was recently shown to interact with Ex and regulate its localization and activity in promoting Hippo activation [34].

**FIGURE 2:**
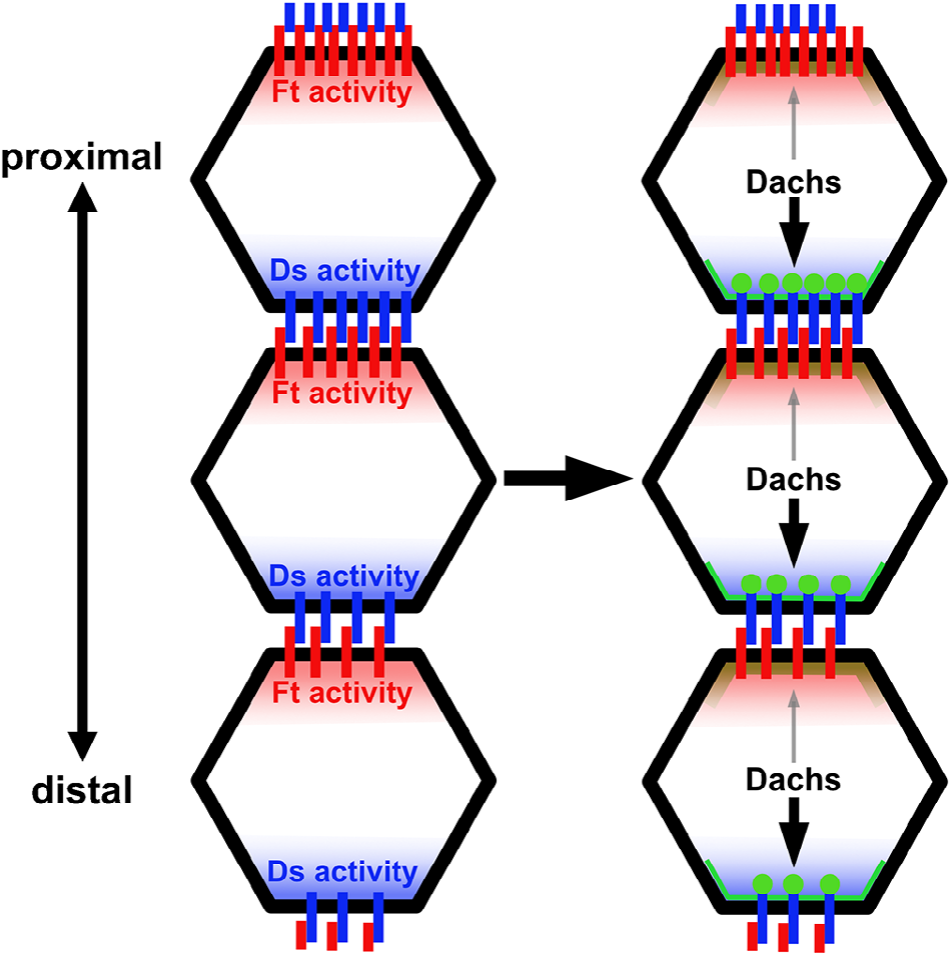
The current model for how planar polarized Ft and Ds activity influences Dachs localization at the junctional cortex. Polarized Ds activity (blue) promotes Dachs accumulation (light green) on the distal side of cells. In contrast, polarized Ft activity (red) prevents Dachs accumulation at the proximal junctions.

The current model for Ds–Ft signaling suggests that Ds promotes growth by recruiting Dachs to the junctional cortex at distal cell edges, thereby promoting Dachs activity (Figure 2). Conversely, Ft represses growth by promoting Dachs degradation at proximal cell edges [18, 21, 32, 33]. However, this model appears inconsistent with the following observations:

1. *ds* and *ft* double mutants exhibit much greater Dachs accumulation and tissue overgrowth than either single mutant, suggesting that Dachs requires neither Ds nor Ft to promote growth and that Ds and Ft synergistically repress growth [35, 36].
2. If Ds is required to recruit Dachs to the junctional cortex, then its loss should lead to the loss of Dachs at the junctional cortex and consequently undergrowth. However, *ds* mutant cells display overgrowth, elevated Dachs expression, and increased accumulation of Dachs at the junctional cortex [26, 31, 36].

In this review, we focus on how the Ds–Ft signaling pathway regulates the abundance, localization, and function of Dachs and its two regulators, Approximated (App) and Dachs ligand with SH3 domains (Dlish) (hereafter referred to as the core complex) at the junctional cortex, as discussed in our recent paper [36]. We propose that the core complex has a default “on” state that strongly promotes tissue growth, even in the absence of both Ds and Ft. Furthermore, while previous models have suggested that Ft and Ds function antagonistically, with Ft repressing growth and Ds promoting it, we propose that Ds and Ft function together to restrict the abundance of the core complex at the junctional cortex, thereby coordinately constraining tissue growth. Because of space constraints and the format of this hypothesis‐based review, we do not attempt to cover all aspects of Ds–Ft signaling and instead refer the reader to recently published reviews on this subject [18, 19, 21, 37].

## 2 Dachs Plays a Key Role in the Ds–Ft Signaling Pathway

Mutations in *dachs* were originally discovered through defects in proximodistal (PD) patterning of appendages. *dachs* encodes an atypical myosin, although it is unknown whether it acts as a motor [38]. Biochemical evidence shows that Dachs does not bind ATP and has no detectable ATPase activity [38]. However, it can bind F‐actin as well as Wts, indicating it may serve as a scaffold to sequester, degrade, or otherwise inactivate Wts [9, 32, 38]. Structural and functional studies revealed that the myosin head domain of Dachs is required for growth control and localization to the junctional cortex [39]. From this result and other data, it is thought that Dachs has to localize to the junctional cortex to promote growth [32].

**FIGURE 3:**
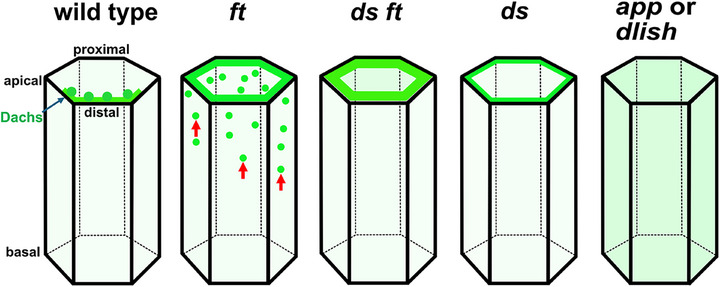
The effects of mutations in Ds–Ft signaling components on Dachs distribution. In wild‐type cells, Dachs (light green) is localized, primarily in distinct puncta, at the junctional cortex on the distal side of the cell (also see Figure 2). In *ft* mutant cells, Dachs is highly enriched at the junctional cortex but lacks puncta and is found in cytoplasmic vesicles that colocalize with Ds [36] (red arrows). In *ds ft* double mutants, Dachs accumulates even more strongly at the junctional cortex but lacks puncta and does not localize to cytoplasmic vesicles. In *ds* mutants, Dachs is enriched at the junctional cortex but not as strongly as in *ft* mutants. In *app* or *dlish* mutants, Dachs fails to accumulate at the junctional cortex and is mislocalized throughout the cytoplasm.

In contrast to the massive overgrowth seen in *ft* mutants, loss of *dachs* leads to severe undergrowth, consistent with the idea that Dachs suppresses Wts activity (which in turn represses Yki) [32]. However, unlike ectopic *wts* activation or *yki* mutants, *dachs* (and *dlish* and *app*) undergrowth phenotypes are relatively mild. This is likely due to redundant cellular mechanisms that repress Wts activity, including the requirement for Crumbs, Ex, Kibra, and Merlin to activate Wts [5, 40, 41], and the ability of Ajuba to bind and sequester Wts in response to junctional tension [42]. Genetic evidence indicates that *dachs* is epistatic to *ft* because removing *dachs* strongly suppresses the overgrowth phenotype of *ft* mutants [32]. Since Dachs accumulates at the junctional cortex and shows increased abundance in *ft* mutants (Figure 3), it is proposed that Ft restricts organ growth by decreasing Dachs levels [32]. Therefore, proper regulation of Dachs localization and abundance is critical for controlling organ growth.

## 3 Approximated and Dlish

Two additional regulators, App and Dlish, are required for Dachs function and for its localization to the junctional cortex [39, 43, 44]. Like *dachs*, *app* mutations produce defects in organ growth and appendage patterning [43]. *app* encodes a DHHC palmitoyltransferase, an enzyme that adds palmitoyl fatty acids to cysteine residues in target proteins. Palmitoylation often affects protein localization or activity by increasing affinity for the plasma membrane [45, 46]. Mutations within the catalytic domain of App cause loss‐of‐function defects, indicating that App does function as a palmitoyltransferase and that this activity is important for its functions in growth control [47]. Like other Ds–Ft signaling components, App is enriched at the junctional cortex [36, 43]. In *app* mutants, Dachs becomes mislocalized throughout the cytoplasm (Figure 3) [43]. Although one might expect that App palmitoylates Dachs to promote its cortical localization, biochemical evidence shows that this is not the case; instead, other regulators, specifically Dlish (see below) and the Ft intracellular domain (ICD) have been shown to be targets for palmitoylation by App [39, 47].

Dlish was identified in a proteomic screen as a Dachs interactor [39, 44]. *dlish* encodes a protein with three SH3 domains, which are known to mediate protein–protein interactions. Loss of *dlish* causes mild undergrowth and PD patterning defects. As in *app* mutants, *dlish* mutants show reduced Dachs at the junctional cortex and mislocalization of Dachs to the cytoplasm (Figure 3) [39, 44]. Dlish itself is greatly reduced in *dachs* mutants, suggesting that Dachs and Dlish are mutually dependent on each other's localization and abundance [39, 44].

## 4 Dachs, Dlish, and App Function Together as a “core complex” That Promotes Growth

How do Dachs, Dlish, and App function in growth control? Genetic evidence shows that removing any one of them suppresses the massive overgrowth in *ft* mutants, suggesting that all three function together to promote growth downstream of Ds and Ft [32, 36, 39, 43, 44]. Previous data have shown that Dachs forms a tight complex with Dlish. Ectopic expression of Dachs results in increased Dlish accumulation, and expression of Dlish similarly increases Dachs levels, suggesting that their ability to form a complex might protect them from proteolytic degradation [39, 44].

**FIGURE 4:**
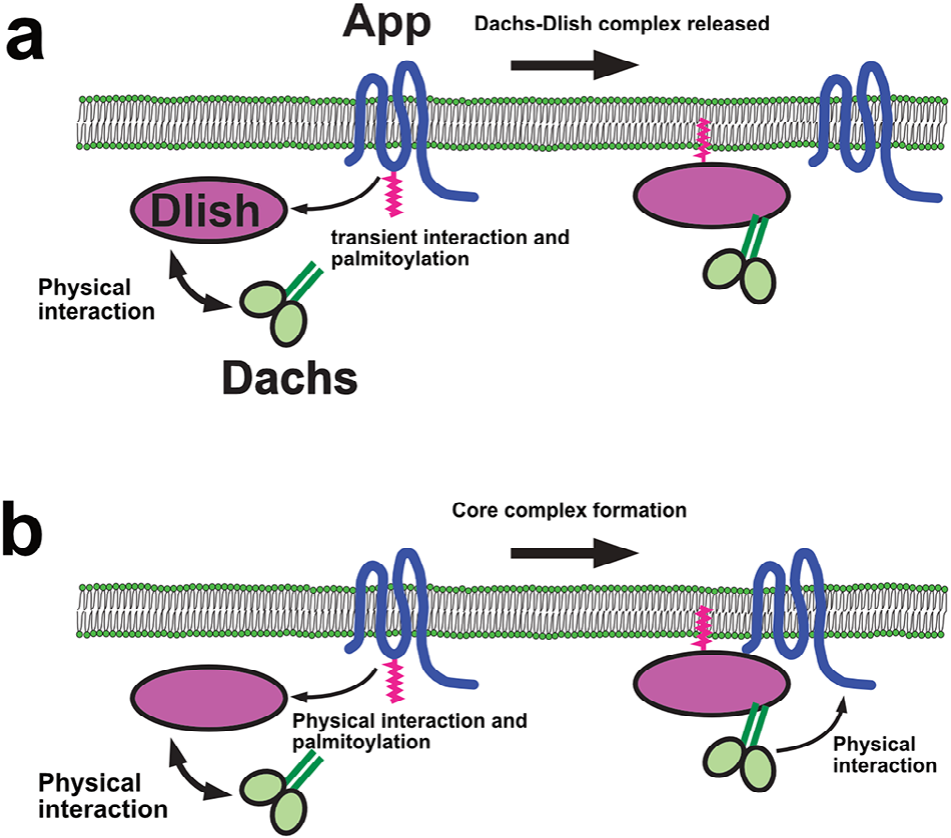
Two possible models for interactions between Dachs, Dlish, and App. In both models, Dachs and Dlish interact stably. In (a), App interacts transiently with Dlish as a substrate for palmitoylation, which promotes the localization of Dlish and Dachs to the cell cortex. In (b), App palmitoylates Dlish but maintains a stable interaction with Dlish and Dachs, stabilizing the core complex through these physical interactions.

In tissues, Dachs and Dlish colocalize with App in junctional puncta, suggesting that App may be a part of the Dachs–Dlish complex [36]. However, because App is a palmitoyltransferase, this interaction might be transient, functioning as an enzyme–substrate complex (Figure 4a). Additionally, although App can interact with both Dlish and Dachs in cultured cells, this interaction seems weaker than the Dachs–Dlish interaction [39, 47]. Thus, it is plausible that App promotes Dachs–Dlish localization at the cell cortex solely through Dlish palmitoylation. However, we have observed that Dachs and Dlish affect the accumulation of App at the junctional cortex [36, 47]. In the absence of Dachs or Dlish, App is significantly reduced, while overexpression of Dachs or Dlish causes increased App accumulation [36, 47]. Additionally, in cultured cells, either Dachs or Dlish can recruit App, and App can recruit them in turn [36]. These observations suggest that these interactions are more stable than expected for an enzyme–substrate interaction (Figure 4b).

Taken together, these observations suggest that Dachs, Dlish, and App form a stable core complex at the junctional cortex that functions to promote tissue growth (Figure 4b). Notably, cortical accumulation of the core complex and tissue growth are far greater in the complete absence of both Ds and Ft than in wild‐type tissues (Figure 3) [36], indicating that the default state of this complex is fully “On” with respect to growth. Furthermore, these observations indicate that Ds and Ft function together to repress the growth‐promoting activity of the core complex [36]. We next address the question of how Ds and Ft repress growth.

## 5 Effects of the Ft ICD on the Core Complex

**FIGURE 5:**
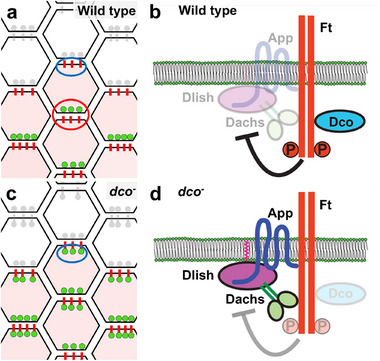
Ft activity prevents localization of Dachs puncta at proximal junctions. To examine co‐localization between Ft and Dachs, clones with or without labeled Dachs and Ft (the two are genetically linked) are generated in a wild‐type or *dco* mutant background. At the boundary between dual‐labeled cells (pale pink hexagons) and unlabeled cells (light grey hexagons), the localization of labeled Ft (red bar) and labeled Dachs (light green circle) can be observed. Proximal is up in (a) and (c). (a) In wild‐type cells, Dachs does not colocalize with Ft puncta at proximal junctions (blue ellipse). Note that within the clone (red ellipse), Ft and Dachs appear to colocalize at puncta due to accumulating Ds (not shown) in the opposing cell. (b) Normally, Dco phosphorylates and activates Ft, preventing accumulation of the core complex. (c) In *dco* mutant cells, Dachs colocalizes with Ft in puncta at proximal junctions (blue ellipse). (d) In the absence of Dco, Ft can recruit the core complex but fails to degrade it. For more experimental details, see [36].

Loss of Ft results in a dramatic increase in Dachs abundance, suggesting that Ft restricts Dachs function by promoting its degradation [32]. How does the Ft ICD do this? The Ft ICD can recruit core complex proteins to the cell cortex in cultured cells and is localized to junctional puncta in imaginal tissues [36]. However, mosaic analysis reveals that in wild‐type cells Ft does not colocalize with core complex proteins in these puncta (Figures 2 and 5a) [36]. A possible explanation for these seemingly contradictory observations is that the Ft ICD recruits the core complex to promote its degradation (Figure 5b). Consistent with this idea, mutations in the *ft* ICD that inactivate its growth function (e.g., *ft^SUM^* or *ft^61^*) and mutations in the kinase that activates Ft, *discs overgrown* (*dco*), result in increased Dachs junctional accumulation and abnormal colocalization of Dachs with Ft in junctional puncta (Figure 5c,d) [36].

Recent work suggests that Ft promotes Dachs degradation in a ubiquitin‐dependent manner [48, 49, 50]. Biochemical evidence indicates that both Dachs and App can be ubiquitinated, though only Dachs abundance has been shown to be increased by loss of Ft [48]. Several genes encoding ubiquitination‐related proteins have been identified as candidates. Fbxl7 encodes a protein with an F‐box and leucine‐rich repeats that interacts with Skp1 and Cul1 to form an SCF E3 ubiquitin ligase complex [49, 50]. Like *ft*, *fbxl7* mutants show mild overgrowth and slightly increased junctional Dachs. Fbxl7 forms a complex with the Ft ICD in cultured cells and colocalizes with Ft at the junctional cortex on the proximal cell edge [49, 50]. However, whether Fbxl7 directly ubiquitinates Dachs in vitro is still debated [49, 50]. Alternatively, Fbxl7 might regulate the trafficking of Ds–Ft pathway proteins more generally and thereby indirectly affect the abundance of Dachs [49].

Two other E3 ubiquitin ligases have been implicated in regulating Dachs abundance. Supernumerary limbs (Slmb) interacts with Dlish and Cul1in cultured cells, but *slmb* mutants display only slightly increased abundance of Dachs and Dlish [39]. This differs markedly from the pronounced increase in Dachs observed in *ft* mutants. *early girl* (*elgi*) encodes a RING domain E3 ubiquitin ligase. *elgi* mutants show mild patterning defects resembling other *ds*–*ft* pathway mutants and display increased junctional Dachs and Dlish [48]. Although Elgi interacts with the Ft ICD, Dachs, and App, it fails to ubiquitinate any of these components [48]. Interestingly, a recent study shows that the de‐ubiquitylating enzyme Fat facets genetically and physically interacts with Ft signaling components [51], further suggesting that ubiquitination plays an important role in Ft–Ds signaling.

Although ubiquitin‐mediated degradation is an appealing model for Dachs regulation, no direct evidence currently exists that Fbxl7, Elgi, or Slmb ubiquitinate Dachs or other pathway components. Additionally, unlike *ft* mutants, *fbxl7* or *elgi* single mutants cause only mild overgrowth (*slmb* mutants have pleiotropic phenotypes due to its many targets). It is possible that a combination of these genes is required for Dachs degradation, or that other, yet unidentified, ubiquitination machinery is involved.

## 6 Effects of the Ds ICD on the Core Complex

**FIGURE 6:**
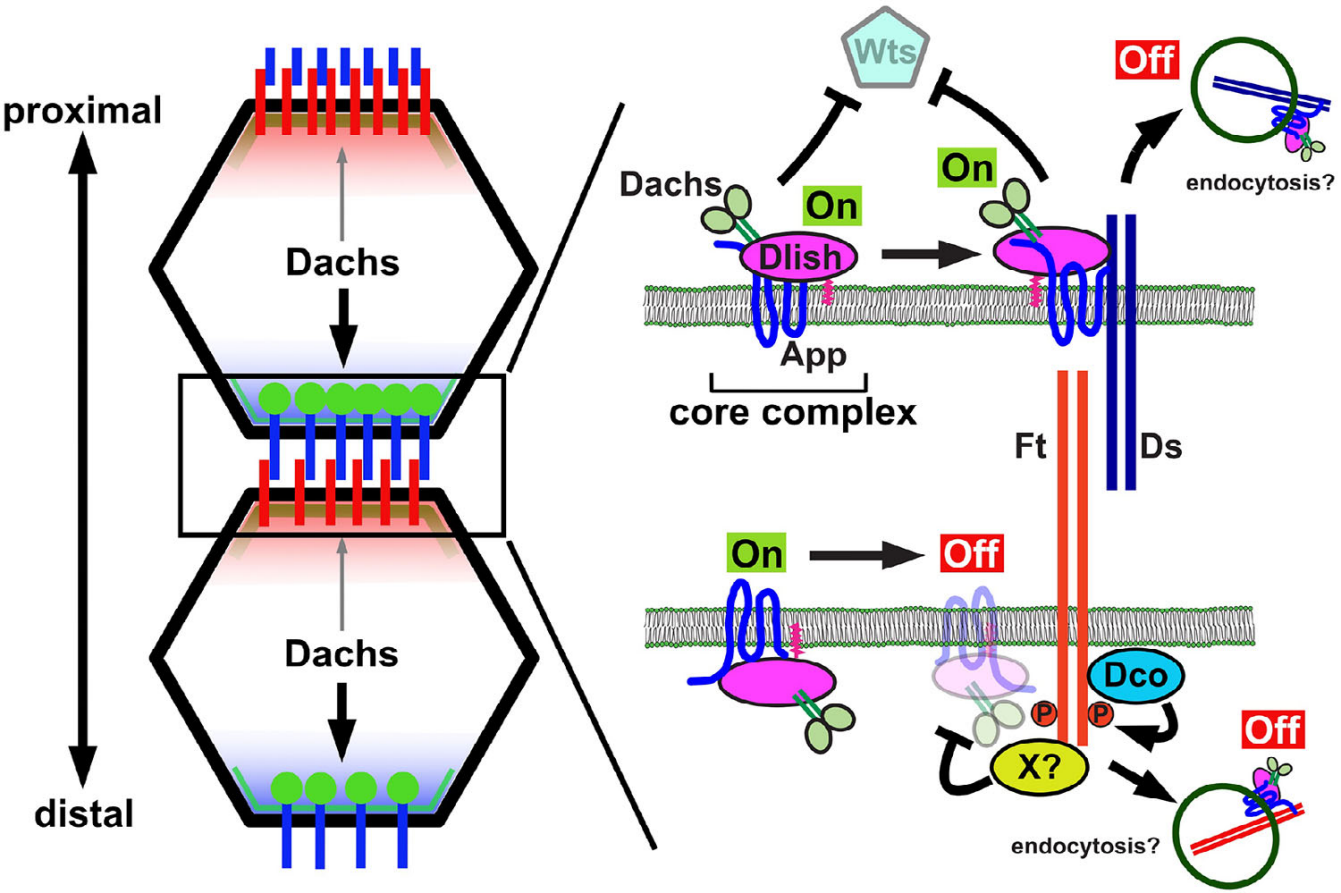
Proposed model for how Ft and Ds coordinately control the core complex and tissue growth. In the absence of Ft or Ds, the core complex accumulates at the junctional cortex at high levels and strongly promotes growth (“On” state). When bound to Ft on adjacent cells, Ds recruits the core complex and stabilizes it at junctional puncta, thereby promoting growth (On state). However, when not bound to Ft, Ds removes the core complex from the junctional cortex, possibly through endocytosis, and thereby restricts growth (“Off” state). Finally, when bound to Ds, Ft recruits the core complex and promotes its degradation through poorly understood processes/proteins, referred to here as protein X. This restricts growth (Off state). Thus, Ds and Ft function coordinately to restrict the activity of the core complex and control growth.

What is the function of the Ds ICD, and how does Ds affect the abundance and localization of the core complex? Studies in cultured cells show that like Ft, the Ds ICD co‐immunoprecipitates with core complex proteins and that the Ds ICD can recruit them to the cell cortex [31, 36, 39, 44, 52]. Unlike Ft, in wild‐type imaginal epithelia the core complex colocalizes with Ds in puncta at the junctional cortex (Figure 2) [36]. However, in the absence of Ft, Ds mislocalizes to cytoplasmic puncta where it colocalizes with Dachs (Figure 3) [36]. Taken together these observations strongly suggest that while Ds is not required for core complex recruitment, the Ds ICD does interact with core complex proteins and can remove them from the junctional cortex, likely through endocytosis (Figure 6) [36]. Consistent with this model, *ds^∆ICD^*, which lacks the Ds ICD, fails to form junctional puncta and does not recruit core complex proteins to cytoplasmic puncta in the absence of Ft [36]. Unexpectedly, *ds^∆ICD^* mutants also exhibit undergrowth. This result suggests that, when Ft is present, a key role for the Ds ICD is to organize core complex proteins into junctional puncta that protect the core complex from Ft‐mediated degradation [36]. In contrast, *ds* null mutants display overgrowth because Ds–Ft binding via their extracellular domains promotes Ft's ability to degrade Dachs [36].

Among the core complex proteins, App seems to be most dependent on Ds for proper subcellular localization. Although loss of Ds results in increased accumulation of Dachs and Dlish, App appears more diffuse and less associated at the junctional cortex in *ds* mutant tissues [36]. Additionally, App accumulates at cell junctions together with Ds but does not do so with Ft [36]. Taken together, these observations suggest that App might interact with Ds more directly than other core complex proteins do.

Comparison of amino acid sequences has identified three Conserved Motifs (CM1–3) in the Ds ICD, shared by *Drosophila* Ds and its human orthologs DCHS1 and DCHS2 [22]. Fine‐scale structure–function studies of the Ds ICD have revealed domains responsible for recruiting or interacting with the core complex, particularly domain D (which overlaps with CM2) as both necessary and sufficient to recruit core complex proteins in cultured cells and in tissues [22, 36, 53]. Although domain D is well conserved, its function in vertebrates is unclear because no Dachs orthologues have been identified. Interestingly, domain D in zebrafish Dchs‐1b interacts with tetratricopeptide repeat‐containing protein 28 (Ttc‐28), which is required for early embryonic development, although the function of Ttc‐28 in *Drosophila* is not yet known [54].

## 7 A Proposed Role for Ds in Repressing Core Complex Function

Signal transduction pathways are often activated or inactivated via receptor‐mediated endocytosis (e.g., the Notch pathway) [55]. We propose that while Ft‐mediated degradation is the main mechanism for down‐regulating the core complex, its removal from the junctional cortex via co‐endocytosis with Ds seems to provide an additional regulatory strategy (Figure 6). Several lines of evidence support this notion. First, although loss of Ft results in severe overgrowth, loss of Ds and Ft together results in far greater overgrowth, indicating that Ds can negatively regulate growth [35, 36]. Second, in the absence of Ft, Ds is found in cytoplasmic puncta that colocalize with core complex proteins (Figure 3) [36]. Additionally, these cells display less junctional Dachs than cells that lack both Ds and Ft [36]. Taken together these observations suggest that Ds and the core complex can be internalized and removed from the junctional cortex (“Off” state), thereby repressing growth (Figure 6). A likely result of this is that Ds and the core complex are trafficked into multi‐vesicular bodies and ultimately degraded in lysosomes, but further studies that examine the relationship of Ds and the core complex with endocytic processes need to be done to test this hypothesis. Ft has similarly been observed to be endocytosed [56], though whether this is a part of ubiquitin‐mediated degradation or separate from it remains unknown.

Although the observations just described clearly suggest that Ds can remove core complex proteins from the junctional cortex to repress growth in the absence of Ft, it remains unclear how important this function is in normal cells. Interestingly, in the proximal wing and hinge, Ds is highly expressed but only binds weakly to Ft due to decreased post‐translational modification by Fj [28, 29]. Thus, it is possible that in the proximal wing, Ds‐mediated removal of the core complex from the junctional cortex is an important contributor to growth control. Careful observation of Ds and the core complex in the proximal wing, combined with manipulation of Fj function, are needed to further test this possibility.

## 8 Conclusions

We propose the following model for how Ds and Ft coordinately regulate the function of the core complex in promoting tissue growth (Figure 6). At the distal junctional cortex, App is restricted within the plasma membrane through interactions with the Ds ICD. Together, Ds and App then promote accumulation of Dachs and Dlish. When Ds is bound to Ft in *trans*, these interactions yield stable accumulations of these proteins into junctional puncta that promote growth. In the absence of binding to Ft, Ds still recruits App, Dlish, and Dachs but instead promotes their removal from the junctional cortex, reducing growth. Ft also recruits core complex proteins at the junctional cortex, but in this case, the interaction is more transient because Dachs is rapidly degraded, and the core complex is inactivated. This leads to asymmetric accumulation of core complex proteins on the distal junctions of cells together with Ds, while Ft accumulates on the proximal junction [36]. In the absence of either Ds or Ft, the core complex accumulates at the junctional cortex at very high levels, demonstrating that neither protein is required for complex formation and resulting in massive overgrowth. These observations reveal that the default state of Dachs and the rest of the core complex is “on,” promoting growth, and that Ft and Ds work together to restrict core complex assembly and growth.

Finally, as with many important biological processes, while much has been learned about the roles of Ds–Ft interactions in tissue growth control, many questions remain to be answered. We note some of the most interesting unanswered questions below.

1. Does Dachs function as a myosin?
  It remains unclear if Dachs functions as a myosin. Dachs binds F‐actin in an ATP‐independent manner but in vitro does not display motor activity, suggesting it may act instead as a scaffold [38]. However, Dachs accumulation correlates positively with junctional tension, suggesting it could have myosin‐like activity [52].
2. How does extracellular Ds–Ft binding affect intracellular signaling?
  Although it is known that Ds‐binding promotes Ft activity in degrading Dachs [36], it is unclear how this occurs. Evidence shows that Ds binding increases Ft ICD phosphorylation in a Dco‐dependent manner [57, 58]. Structural analyses using AlphaFold may help clarify how Ds–Ft interaction affects the conformation of their intracellular domains.
3. What is the function of Ds–Ft interactions in *cis*, particularly between their ICDs?
  Previous work has shown that Ft and Ds co‐exist in junctional puncta, though Ds predominates on distal junctions and Ft predominates on proximal ones due to the planar polarized interactions described earlier [59]. Additionally, the Ft and Ds ICDs can physically interact [34] and ectopic expression of the Ft ICD can reduce Ds in tissues [50]. This result suggests that Ft could promote the degradation of Ds *in cis*, thereby contributing to their planar polarized distribution in tissues. Conversely, because App palmitoylates the Ft ICD and suppresses Ft activity [47], an interesting possibility is that in predominantly Ds puncta the high levels of App recruited by Ds could suppress Ft activity. Whether Ds–Ft *cis*‐interactions have other functions and how this might contribute to growth control remains to be established.
4. What is the relationship between the functions of Ds and Ft in flies and mammals?
  Although the intracellular domains of Ft and Ds are highly conserved between *Drosophila* and mammals, it is unclear that their functions in growth control are well conserved. So far, a “core complex” that functions downstream of mammalian Ds and Ft has not been identified. Further studies of the functions of these highly conserved domains in both flies and mammals will be required to better understand why they are conserved.
5. What is the functional significance of core complex puncta at the junctional cortex?
  We know that the localization of core complex components in junctional puncta depends on Ds and Ft, but we do not fully understand how they form or their importance in growth control. Do they simply protect core complex components from Ft‐mediated degradation, or do they modulate the activity of this complex in other ways?

## Author Contributions

H.M. wrote the original draft of this manuscript which was then edited by H.M. and R.G.F.

## Conflicts of Interest

The authors declare no conflicts of interest.

## Data Availability

Data sharing is not applicable to this article as no datasets were generated or analyzed for this article.

## References

[1] M. I. Worley , L. Setiawan , and I. K. Hariharan , “TIE‐DYE: A Combinatorial Marking System to Visualize and Genetically Manipulate Clones During Development in *Drosophila Melanogaster* ,” Development (Cambridge, England) 140, no. 15 (2013): 3275–3284, 10.1242/dev.096057. 23785055 PMC3931737

[2] M. Mandaravally Madhavan and H. A. Schneiderman , “Histological Analysis of the Dynamics of Growth of Imaginal Discs and Histoblast Nests During the Larval Development of Drosophila melanogaster,” Wilhelm Roux's Archives of Developmental Biology 183, no. 4 (1977): 269–305, 10.1007/BF00848459. 28304865

[3] A. Kicheva and J. Briscoe , “Control of Tissue Development by Morphogens,” Annual Review of Cell and Developmental Biology 39, no. 1 (2023): 91–121, 10.1146/annurev-cellbio-020823-011522. 37418774

[4] J. R. Misra and K. D. Irvine , “The Hippo Signaling Network and Its Biological Functions,” Annual Review of Genetics 52, no. 1 (2018): 65–87, 10.1146/annurev-genet-120417-031621. PMC6322405 30183404

[5] Y. Zheng and D. Pan , “The Hippo Signaling Pathway in Development and Disease,” Developmental Cell 50, no. 3 (2019): 264–282, 10.1016/j.devcel.2019.06.003. 31386861 PMC6748048

[6] R. Karaman and G. Halder , “Cell Junctions in Hippo Signaling,” Cold Spring Harbor Perspectives in Biology 10, no. 5 (2018): a028753, 10.1101/cshperspect.a028753. 28600393 PMC5932588

[7] J. C. Boggiano and R. G. Fehon , “Growth Control by Committee: Intercellular Junctions, Cell Polarity, and the Cytoskeleton Regulate Hippo Signaling,” Developmental Cell 22, no. 4 (2012): 695–702, 10.1016/j.devcel.2012.03.013. 22516196 PMC3376383

[8] T. Su , M. Z. Ludwig , J. Xu , and R. G. Fehon , “Kibra and Merlin Activate the Hippo Pathway Spatially Distinct From and Independent of Expanded,” Developmental Cell 40, no. 5 (2017): 478–490, 10.1016/j.devcel.2017.02.004. 28292426 PMC5414729

[9] E. Cho , Y. Feng , C. Rauskolb , S. Maitra , R. Fehon , and K. D. Irvine , “Delineation of a Fat Tumor Suppressor Pathway,” Nature Genetics 38, no. 10 (2006): 1142–1150, 10.1038/ng1887. 16980976

[10] A. M. Vrabioiu and G. Struhl , “Fat/Dachsous Signaling Promotes Drosophila Wing Growth by Regulating the Conformational State of the NDR Kinase Warts,” Developmental Cell 35, no. 6 (2015): 737–749, 10.1016/j.devcel.2015.11.027. 26702832 PMC4709125

[11] M. Willecke , F. Hamaratoglu , M. Kango‐Singh , et al., “The Fat Cadherin Acts Through the Hippo Tumor‐Suppressor Pathway to Regulate Tissue Size,” Current Biology 16, no. 21 (2006): 2090–2100, 10.1016/j.cub.2006.09.005. 16996265

[12] E. Silva , Y. Tsatskis , L. Gardano , N. Tapon , and H. McNeill , “The Tumor‐Suppressor Gene Fat Controls Tissue Growth Upstream of Expanded in the Hippo Signaling Pathway,” Current Biology 16, no. 21 (2006): 2081–2089, 10.1016/j.cub.2006.09.004. 16996266

[13] F. C. Bennett and K. F. Harvey , “Fat Cadherin Modulates Organ Size in Drosophila via the Salvador/Warts/Hippo Signaling Pathway,” Current Biology 16 (2006): 2101–2110, 10.1016/j.cub.2006.09.045. 17045801

[14] P. A. Mahoney , U. Weber , P. Onofrechuk , H. Biessmann , P. J. Bryant , and C. S. Goodman , “The Fat Tumor Suppressor Gene in Drosophila Encodes a Novel Member of the Cadherin Gene Superfamily,” Cell 67, no. 5 (1991): 853–868, 10.1016/0092-8674(91)90359-7. 1959133

[15] H. F. Clark , D. Brentrup , K. Schneitz , A. Bieber , C. Goodman , and M. Noll , “Dachsous Encodes a Member of the Cadherin Superfamily That Controls Imaginal Disc Morphogenesis in Drosophila,” Genes & Development 9, no. 12 (1995): 1530–1542, 10.1101/gad.9.12.1530. 7601355

[16] P. J. Bryant , B. Huettner , L. I. Held , J. Ryerse , and J. Szidonya , “Mutations at the Fat Locus Interfere With Cell Proliferation Control and Epithelial Morphogenesis in Drosophila,” Developmental Biology 129, no. 2 (1988): 541–554, 10.1016/0012-1606(88)90399-5. 3417051

[17] P. N. Adler , J. Charlton , and J. Liu , “Mutations in the Cadherin Superfamily Member Gene *Dachsous* Cause a Tissue Polarity Phenotype by Altering *Frizzled* Signaling,” Development (Cambridge, England) 125, no. 5 (1998): 959–968, 10.1242/dev.125.5.959. 9449678

[18] S. Blair and H. McNeill , “Big Roles for Fat Cadherins,” Current Opinion in Cell Biology 51 (2018): 73–80, 10.1016/j.ceb.2017.11.006. 29258012 PMC5949260

[19] H. Strutt and D. Strutt , “How Do the Fat–Dachsous and Core Planar Polarity Pathways Act Together and Independently to Coordinate Polarized Cell Behaviours?,” Open Biology 11, no. 2 (2021): 200356, 10.1098/rsob.200356. 33561385 PMC8061702

[20] P. A. Lawrence , G. Struhl , and J. Casal , “Do the Protocadherins Fat and Dachsous Link up to Determine both Planar Cell Polarity and the Dimensions of Organs?,” Nature Cell Biology 10, no. 12 (2008): 1379–1382, 10.1038/ncb1208-1379. 19043429 PMC2747020

[21] A. Gridnev and J. R. Misra , “Emerging Mechanisms of Growth and Patterning Regulation by Dachsous and Fat Protocadherins,” Frontiers in Cell and Developmental Biology 10 (2022): 842593, 10.3389/fcell.2022.842593. 35372364 PMC8967653

[22] P. Hulpiau and F. Van Roy , “Molecular Evolution of the Cadherin Superfamily,” International Journal of Biochemistry & Cell Biology 41, no. 2 (2009): 349–369, 10.1016/j.biocel.2008.09.027. 18848899

[23] H. Matakatsu and S. S. Blair , “Interactions Between Fat and Dachsous and the Regulation of Planar Cell Polarity in the *Drosophila Wing* ,” Development (Cambridge, England) 131, no. 15 (2004): 3785–3794, 10.1242/dev.01254. 15240556

[24] D. Ma , C. Yang , H. McNeill , M. A. Simon , and J. D. Axelrod , “Fidelity in Planar Cell Polarity Signalling,” Nature 421, no. 6922 (2003): 543–547, 10.1038/nature01366. 12540853

[25] H. Strutt and D. Strutt , “Nonautonomous Planar Polarity Patterning in Drosophila,” Developmental Cell 3, no. 6 (2002): 851–863, 10.1016/S1534-5807(02)00363-5. 12479810

[26] A. Brittle , C. Thomas , and D. Strutt , “Planar Polarity Specification Through Asymmetric Subcellular Localization of Fat and Dachsous,” Current Biology 22, no. 10 (2012): 907–914, 10.1016/j.cub.2012.03.053. 22503504 PMC3362735

[27] A. A. Ambegaonkar , G. Pan , M. Mani , Y. Feng , and K. D. Irvine , “Propagation of Dachsous‐Fat Planar Cell Polarity,” Current Biology 22, no. 14 (2012): 1302–1308, 10.1016/j.cub.2012.05.049. 22727698 PMC3418676

[28] A. L. Brittle , A. Repiso , J. Casal , P. A. Lawrence , and D. Strutt , “Four‐Jointed Modulates Growth and Planar Polarity by Reducing the Affinity of Dachsous for Fat,” Current Biology 20, no. 9 (2010): 803–810, 10.1016/j.cub.2010.03.056. 20434337 PMC2958304

[29] M. A. Simon , A. Xu , H. O. Ishikawa , and K. D. Irvine , “Modulation of Fat:Dachsous Binding by the Cadherin Domain Kinase Four‐Jointed,” Current Biology 20, no. 9 (2010): 811–817, 10.1016/j.cub.2010.04.016. 20434335 PMC2884055

[30] H. O. Ishikawa , H. Takeuchi , R. S. Haltiwanger , and K. D. Irvine , “Four‐Jointed Is a Golgi Kinase That Phosphorylates a Subset of Cadherin Domains,” Science (New York, N.Y.) 321, no. 5887 (2008): 401–404, 10.1126/science.1158159. PMC2562711 18635802

[31] F. Bosveld , I. Bonnet , B. Guirao , et al., “Mechanical Control of Morphogenesis by Fat/Dachsous/Four‐Jointed Planar Cell Polarity Pathway,” Science (New York, N.Y.) 336, no. 6082 (2012): 724–727, 10.1126/science.1221071. 22499807

[32] Y. Mao , C. Rauskolb , E. Cho , et al., “Dachs: An Unconventional Myosin That Functions Downstream of Fat to Regulate Growth, Affinity and Gene Expression in *Drosophila* ,” Development (Cambridge, England) 133, no. 13 (2006): 2539–2551, 10.1242/dev.02427. 16735478

[33] D. Rogulja , C. Rauskolb , and K. D. Irvine , “Morphogen Control of Wing Growth Through the Fat Signaling Pathway,” Developmental Cell 15, no. 2 (2008): 309–321, 10.1016/j.devcel.2008.06.003. 18694569 PMC2613447

[34] A. D. Fulford , L. Enderle , J. Rusch , et al., “Expanded Directly Binds Conserved Regions of Fat to Restrain Growth via the Hippo Pathway,” Journal of Cell Biology 222, no. 5 (2023): 202204059, 10.1083/jcb.202204059. PMC10120405 37071483

[35] H. Matakatsu and S. S. Blair , “Separating the Adhesive and Signaling Functions of the Fat and Dachsous Protocadherins,” Development (Cambridge, England) 133, no. 12 (2006): 2315–2324, 10.1242/dev.02401. 16687445

[36] H. Matakatsu and R. G. Fehon , “Dachsous and Fat Coordinately Repress the Dachs–Dlish–Approximated Complex to Control Growth,” Journal of Cell Biology 223, no. 12 (2024): 202406119, 10.1083/jcb.202406119. PMC11461286 39373700

[37] J. Kasiah and H. McNeill , “Fat and Dachsous Cadherins in Mammalian Development,” Current Topics in Developmental Biology 154 (2023): 223–244, 10.1016/bs.ctdb.2023.02.008. 37100519

[38] Y. Cao , H. D. White , and X. Li , “ *Drosophila* Myosin‐XX Functions as an Actin‐Binding Protein to Facilitate the Interaction Between Zyx102 and Actin,” Biochemistry 53, no. 2 (2014): 350–360, 10.1021/bi401236c. 24393048

[39] Y. Zhang , X. Wang , H. Matakatsu , R. Fehon , and S. S. Blair , “The Novel SH3 Domain Protein Dlish/CG10933 Mediates Fat Signaling in Drosophila by Binding and Regulating Dachs,” Elife 5 (2016): 16624, 10.7554/eLife.16624. PMC5047748 27692068

[40] S. Sun , B. V. V. G. Reddy , and K. D. Irvine , “Localization of Hippo Signalling Complexes and Warts Activation in Vivo,” Nature Communications 6 (2015): 8402, 10.1038/ncomms9402. PMC4598633 26420589

[41] T. Su , M. Z. Ludwig , J. Xu , and R. G. Fehon , “Kibra and Merlin Activate the Hippo Pathway Spatially Distinct From and Independent of Expanded,” Developmental Cell 40, no. 5 (2017): 478–490.e3, 10.1016/j.devcel.2017.02.004. 28292426 PMC5414729

[42] C. Rauskolb , S. Sun , G. Sun , Y. Pan , and K. D. Irvine , “Cytoskeletal Tension Inhibits Hippo Signaling Through an Ajuba‐Warts Complex,” Cell 158, no. 1 (2014): 143–156, 10.1016/j.cell.2014.05.035. 24995985 PMC4082802

[43] H. Matakatsu and S. S. Blair , “The DHHC Palmitoyltransferase Approximated Regulates Fat Signaling and Dachs Localization and Activity,” Current Biology 18, no. 18 (2008): 1390–1395, 10.1016/j.cub.2008.07.067. 18804377 PMC2597019

[44] J. R. Misra and K. D. Irvine , “Vamana Couples Fat Signaling to the Hippo Pathway,” Developmental Cell 39, no. 2 (2016): 254–266, 10.1016/j.devcel.2016.09.017. 27746048 PMC5102026

[45] M. E. Linder and R. J. Deschenes , “Palmitoylation: Policing Protein Stability and Traffic,” Nature Reviews Molecular Cell Biology 8, no. 1 (2007): 74–84, 10.1038/nrm2084. 17183362

[46] Y. Fukata and M. Fukata , “Protein Palmitoylation in Neuronal Development and Synaptic Plasticity,” Nature Reviews Neuroscience 11, no. 3 (2010): 161–175, 10.1038/nrn2788. 20168314

[47] H. Matakatsu , S. S. Blair , and R. G. Fehon , “The Palmitoyltransferase Approximated Promotes Growth via the Hippo Pathway by Palmitoylation of Fat,” Journal of Cell Biology 216, no. 1 (2017): 265–277, 10.1083/jcb.201609094. 28031421 PMC5223609

[48] J. R. Misra and K. D. Irvine , “Early Girl Is a Novel Component of the Fat Signaling Pathway,” PLOS Genetics 15, no. 1 (2019): 1007955, 10.1371/journal.pgen.1007955. PMC6370246 30699121

[49] J. A. Bosch , T. M. Sumabat , Y. Hafezi , B. J. Pellock , K. D. Gandhi , and I. K. Hariharan , “The Drosophila F‐Box Protein Fbxl7 Binds to the Protocadherin Fat and Regulates Dachs Localization and Hippo Signaling,” Elife 3 (2014): 03383, 10.7554/eLife.03383. PMC4144329 25107277

[50] M. Rodrigues‐Campos and B. J. Thompson , “The Ubiquitin Ligase FbxL7 Regulates the Dachsous‐Fat‐Dachs System in *Drosophila* ,” Development (Cambridge, England) 141, no. 21 (2014): 4098–4103, 10.1242/dev.113498. 25256343 PMC4302899

[51] L. E. Dawson , A. Sekar , A. D. Fulford , R. I. Lambert , H. S. Burgess , and P. S. Ribeiro , “The Deubiquitylating Enzyme Fat Facets Promotes Fat Signalling and Restricts Tissue Growth,” Nature Communications 16, no. 1 (2025): 1938, 10.1038/s41467-025-57164-3. PMC11850632 39994229

[52] F. Bosveld , B. Guirao , Z. Wang , et al., “Modulation of Junction Tension by Tumor‐Suppressors and Proto‐Oncogenes Regulates Cell‐Cell Contacts,” Development (Cambridge, England) 143, no. 4 (2016): 623–634, http://dev.biologists.org/lookup/doi/10.1242/dev.127993. 26811379 10.1242/dev.127993

[53] B. K. Tripathi and K. D. Irvine , “Contributions of the Dachsous Intracellular Domain to Dachsous‐Fat Signaling,” Development (Cambridge, England) 151, no. 23 (2024): dev202919, 10.1242/dev.202919. 39503213 PMC11634027

[54] J. Chen , G. D. Castelvecchi , N. Li‐Villarreal , et al., “Atypical Cadherin Dachsous1b Interacts With Ttc28 and Aurora B to Control Microtubule Dynamics in Embryonic Cleavages,” Developmental Cell 45, no. 3 (2018): 376–391, 10.1016/j.devcel.2018.04.009. 29738714 PMC5983389

[55] S. Yamamoto , W.‐L. Charng , and H. J. Bellen , “Endocytosis and Intracellular Trafficking of Notch and Its Ligands,” Current Topics in Developmental Biology 92 (2010): 165–200, 10.1016/s0070-2153(10)92005-x. 20816395 PMC6233319

[56] R. Hale , A. L. Brittle , K. H. Fisher , N. A. M. Monk , and D. Strutt , “Cellular Interpretation of the Long‐Range Gradient of Four‐Jointed Activity in the Drosophila Wing,” Elife 4 (2015): 05789, 10.7554/eLife.05789. PMC4338440 25707557

[57] R. Sopko , E. Silva , L. Clayton , et al., “Phosphorylation of the Tumor Suppressor Fat Is Regulated by Its Ligand Dachsous and the Kinase Discs Overgrown,” Current Biology 19, no. 13 (2009): 1112–1117, 10.1016/j.cub.2009.05.049. 19540118 PMC2851237

[58] Y. Feng and K. D. Irvine , “Processing and Phosphorylation of the Fat Receptor,” Proceedings of the National Academy of Sciences of the United States of America 106, no. 29 (2009): 11989–11994, 10.1073/pnas.0811540106. 19574458 PMC2709664

[59] A. Brittle , S. J. Warrington , H. Strutt , E. Manning , S. E. Tan , and D. Strutt , “Distinct Mechanisms of Planar Polarization by the Core and Fat‐Dachsous Planar Polarity Pathways in the Drosophila Wing,” Cell Reports 40, no. 13 (2022): 111419, 10.1016/j.celrep.2022.111419. 36170824 PMC9631118

